# Flies from meat processing facilities are carriers of multidrug-resistant *Escherichia coli* and diverse *Staphylococcaceae* species

**DOI:** 10.1007/s42770-026-01883-2

**Published:** 2026-02-23

**Authors:** Matheus Zorzal Bernardes Rangel, Gustavo Guimarães Fernandes Viana, Ana Julia Camuzzi Ferrari Storck, Carolina Magri Ferraz, Sarah Bernardes Simões, Valéria Modolo Peterle, Natalia Pereira, Pamella Almeida Freire Casemiro, Alessandra Figueiredo de Castro Nassar, Vanessa Castro, Bruna Maria Salotti-Souza, Juliano Gonçalves Pereira, Marita Vedovelli Cardozo, Gabriel Augusto Marques Rossi

**Affiliations:** 1 Department of Veterinary Medicine, University Vila Velha (UVV), Vila Velha, ES 29102-920 Brazil; 2 Department of Animal Production and Preventive Veterinary Medicine, School of Veterinary Medicine and Animal Science, São Paulo State University (UNESP), Botucatu, 18618-681 SP Brazil; 3 Frigovix Slaughterhouse, Rodovia do Sol, km 86, Anchieta, 29230-000 Espírito Santo Brazil; 4 Department of Pathology, Reproduction and One Health, Faculty of Agricultural and Veterinary Sciences, São Paulo State University, Jaboticabal, São Paulo 14884-900 Brazil; 5 Instituto Biológico (IB) de São Paulo, Rua Conselheiro Rodrigues Alves 1252, São Paulo, São Paulo 04014-002 Brazil; 6 Department of Technology and Inspection of Products of Animal Origin, School of Veterinary Medicine, Universidade Federal de Minas Gerais, Belo Horizonte, Minas Gerais 31270-901 Brazil

**Keywords:** Abattoir, Antimicrobial resistance, Enterobacteriaceae, Food safety, MDR, Vectors

## Abstract

Flies are important vectors of antimicrobial resistance (AMR) in food production, but their role in meat processing environments remains poorly characterized. This study isolated *Enterobacteriaceae* and *Staphylococcaceae* (including *Mammaliicoccus sciuri*) from flies collected in bovine and swine slaughterhouses and meat processing industries. Phenotypic AMR was assessed, and the presence of selected antimicrobial resistance and virulence genes (*mecA*,* sea*,* see*,* sec*,* stx1*,* stx2*, and *eae*) was verified. Out of 60 flies collected, a total of 34 isolates were identified using MALDI-TOF with 13 different bacterial species, including *Escherichia coli*,* Citrobacter braakii*,* C. freundii*,* Klebsiella pneumoniae*,* Hafnia alvei*,* Enterobacter kobei*,* S. aureus*,* S. epidermidis*,* S. saprophyticus*,* S. simulans*,* S. warneri*,* S. xylosus*, and *M. sciuri*. Among *Staphylococcaceae* isolates, 17.6% were resistant to oxacillin and 23.5% to gentamicin. Regarding the *Enterobacteriaceae* isolates, 41.2% were resistant to streptomycin. Ten isolates (two *E. coli*, one *S*. *epidermidis*, two *S. saprophyticus*, one *S. simulans*, one *S*. *warneri* and three *M. sciuri*) were considered multidrug-resistant (MDR). Notably, none of the targeted genes (*mecA*, *sea*, *see*, *sec*, *stx1*, *stx2*, *eae*) were detected by PCR. These findings suggest that flies can disseminate important resistant opportunistic bacteria in meat processing environments, posing concerns due to the capacity of flies to transmit these bacteria across food, processing facilities, animals and humans. These results underscore the need for continuous AMR surveillance and effective vector control to reduce the risks posed by flies in meat processing environments.

**Supplementary Information:**

The online version contains supplementary material available at 10.1007/s42770-026-01883-2.

## Introduction

Although global efforts have been made to reduce antimicrobial misuse, antimicrobial resistance (AMR) continues to spread among both pathogenic and commensal microorganisms [77]. AMR is one of the most critical global public health challenges, with projections estimating up to 8 million deaths annually by 2050 [50] and an associated economic burden exceeding US$1 trillion per year [2]. A comprehensive understanding of AMR is therefore essential, particularly regarding the role of insects such as flies [13, 14]. In low- and middle-income countries, including Brazil, the problem is further aggravated by tropical climates that favor flies’ population growth [26].

Flies are dipteran insects possessing a single wing pair used for flight and a second pair, small knob-like structures that assist with balance during movement [87]. Because of their synanthropic behavior and their coprophagic and saprophagous feeding habits, flies frequently come into contact with decaying organic matter, animal excreta, and food waste [59]. Flies display behaviors and biological traits that increase their exposure to bacteria [55], particularly when they inhabit contaminated environments that are crucial for the development and survival of their offspring [51]. The distinctive physical features of flies, such as sticky leg pads, dense body hairs, electrostatically charged exoskeletons, and sponging mouthparts, facilitate the efficient acquisition and transmission of bacteria [37, 69]. During foraging, flies acquire pathogens both on their external surfaces and by ingesting contaminated fluids [41]. Female flies often carry higher bacterial loads, mainly because they frequent heavily contaminated oviposition sites [53]. Their high mobility, with some species traveling up to 20 km over their lifetime, further enhances their role in bacterial dissemination [39]. Moreover, the ingestion of flies by insectivores may represent an additional route of bacterial spread [58].

In slaughterhouses, flies are in constant contact with primary sources of antimicrobial-resistant bacteria, such as the animals, feces, and waste [16, 56] and can subsequently transmit these bacteria to meat intended for human consumption or human workers. Flies disseminate antimicrobial-resistant pathogens through both mechanical and biological pathways [60] and may contribute to the horizontal transfer of antimicrobial resistance genes, often mediated by mobile genetic elements such as plasmids and transposons [69]. Several studies have demonstrated plasmid-mediated horizontal transfer of resistance genes, including those conferring cephalosporin and tetracycline resistance, within the fly gut from donor to recipient bacteria [33, 34, 60, 80]. These resistant bacteria can persist as part of the fly gut microbiota throughout its lifespan of one to three months [89], thereby sustaining continuous contamination [79].

The most common flies in Brazilian meat production are the housefly, *Musca domestica* L. (Diptera: Muscidae) [6, 20, 81] and the stable fly *Stomoxys calcitrans* (Diptera: Muscidae) [11]. Although this study did not determine the species of the collected flies, these two Diptera are consistently reported as the dominant synanthropic species in slaughterhouses and meat-processing facilities and are therefore the most likely vectors contributing to bacterial acquisition and dissemination in these settings.

Recently, multidrug-resistant (MDR) *E. coli* has been isolated from flies in slaughterhouses [16]. *E. coli* is a major indicator organism for fecal contamination and AMR surveillance in food-producing environments [8, 72], and is one of the leading causes of clinical infection worldwide [61]. The increasing detection of MDR *E. coli* in food-producing animals [63] heightens concern, since they can limit therapeutic options and facilitate the movement of clinically important resistance determinants along the food chain. Within *Staphylococcaceae*, *S. aureus* remains a major zoonotic pathogen, circulating between animals, workers, insects and food products [49, 56]. Coagulase-negative staphylococci also warrant attention, as they increasingly function as reservoirs for AMR and virulence determinants [36]; notably, [13, 14] reported a 53.1% prevalence of MDR in coagulase-negative staphylococci recovered from flies in animal farms.

These attributes highlight flies as valuable organisms for environmental monitoring, particularly in the context of AMR [86]. Their wide distribution, combined with the ease and low cost of capture and processing, makes them ideal sentinels for AMR surveillance programs [87]. Monitoring the microbial communities associated with houseflies thus provides a useful approach for detecting potential microbial threats in the environment [65].

Thus, the present study aimed to isolate and characterize bacteria from flies collected in Brazilian slaughterhouses and meat-processing facilities, focusing on *Staphylococcaceae* and *Enterobacteriaceae*, to evaluate their antimicrobial resistance profiles and investigate the presence of key resistance and virulence genes. This work contributes to addressing an important knowledge gap regarding the role of flies as reservoirs and disseminators of AMR bacteria in food production environments.

## Materials and methods

### Sampling

Flies were collected between September and November of 2024 from five meat production facilities, including two bovine slaughterhouses (A and B), one swine slaughterhouse (C), and two meat processing industries (D and E), located in the cities of Anchieta, Cariacica, Vila Velha and Viana in Espírito Santo state, Brazil.

Flies were directly captured using sterile flasks when they landed on surfaces within the facilities. The flies were sacrificed within two hours after capture by freezing in commercial freezers at −18 ± 2 °C. The samples were processed within 24 h after collection. Sampling sites included indoor areas of slaughterhouses, where animal slaughter was performed, and meat processing plants, where meat industrialization took place. A total of 60 flies were collected from different sites of meat processing facilities. A set of 26 samples were collected from two bovine slaughterhouses (11 and 15 flies coming from the slaughterhouses A and B). Five flies came from the swine slaughterhouse (C). The remaining 29 flies were obtained from two different meat processing industries (14 and 15 flies from meat processing industries D and E).

### Bacterial isolation and identification by MALDI-TOF MS

The collected flies were first transferred to sterile tubes containing Brain Heart Infusion broth (BHI) (Kasvi^®^, Spain) and incubated at 37 °C for 24 h and subsequently agitated using a Vortex. After incubation, 0.1 mL of this broth was plated into Mannitol Salt Agar (MSA) (Kasvi^®^, Spain) for isolation of *Staphylococcus* spp. and Eosin Methylene Blue Agar (EMB) (Kasvi^®^, Spain) for isolation of *E. coli*, with posterior incubation at 37 °C for 24 h. The MSA colonies were considered presumptive for *Staphylococcus* spp. when they exhibited whitish, grayish, or slightly translucent colonies with the medium remaining red-pink, or colonies suggestive of *S. aureus* with a yellowish coloration and a shift of the medium to yellow [21]. The EMB colonies were considered presumptive of *E. coli* when they exhibited a metallic green sheen.

Presumptive colonies were identified using matrix-assisted laser desorption/ionization–time-of-flight mass spectrometry (MALDI-TOF MS) [32] (Microflex LT/SH system, Bruker, Billerica, MA, USA). All isolates were preserved in BHI broth with 30% glycerol on −18 ± 2 °C.

### Antimicrobial susceptibility testing

The disk-diffusion test was used to evaluate the phenotypic AMR profile of the isolates according to [12]. The isolates were prepared by inoculating them onto nutrient agar (Kasvi^®^, Curitiba, Brazil) and incubating at 37 °C for 24 h to prepare the inoculum for the disk-diffusion test. The inoculum was prepared in tubes containing 3 mL of 0.85% saline solution, with the solution being turbidified with the culture until reaching an OD625 nm of 0.1 to 0.2 (0.5 scale MacFarland’s standard). Subsequently, these inoculums were streaked onto plates containing Mueller–Hinton Agar (Kasvi^®^, Spain) using a sterile swab, and disks containing selected antimicrobial agents were placed on the plates.

For the *Enterobacteriaceae* isolates, a total of 16 antimicrobials from 10 different categories were used. The β-lactam/β-lactamase inhibitor combination group included amoxicillin-clavulanic acid (AMC, 30 µg). The monobactams were represented by aztreonam (ATM, 30 µg). Carbapenems tested were imipenem (IMP, 10 µg) and meropenem (MER, 10 µg). The cephalosporin used included the third-generation ceftazidime (CAZ, 30 µg) and ceftriaxone (CRO, 30 µg), and the fourth-generation cefepime (CPM, 30 µg). Aminoglycosides were streptomycin (EST, 10 µg) and gentamicin (GEN, 10 µg). Tetracyclines tested were doxycycline (DOX, 30 µg) and tetracycline (TET, 10 µg). The quinolone group included ciprofloxacin (CIP, 5 µg) and norfloxacin (NOR, 10 µg). Other agents were trimethoprim-sulfamethoxazole (SUT, 25 µg) for folate pathway inhibitors, chloramphenicol (CLO, 30 µg) for phenicols, and nitrofurantoin (NIT, 300 µg) for nitrofurans.

For *Staphylococcaceae* isolates, 18 antimicrobials from 11 different categories were used. The penicillins were ampicillin (AMP, 10 µg) and penicillin (PEN, 10 UI). The β-lactam antibiotics tested included the penicillinase-resistant penicillin oxacillin (OXA, 1 µg) and the second-generation cephalosporin cefoxitin (CFO, 30 µg). Aminoglycosides were amikacin (AMI, 30 µg) and gentamicin (GEN, 10 µg). Tetracyclines tested were doxycycline (DOX, 30 µg) and tetracycline (TET, 10 µg). The quinolone group included enrofloxacin (ENO, 5 µg), levofloxacin (LEV, 5 µg), and moxifloxacin (MFX, 5 µg). The folate pathway inhibitor used was trimethoprim-sulfamethoxazole (SUT, 25 µg). Chloramphenicol (CLO, 30 µg) was used for phenicols. The nitrofurans were represented by nitrofurantoin (NIT, 300 µg). The rifamycin used was rifampicin (RIF, 5 µg). The lincosamide was clindamycin (CLI, 2 µg). Finally, the macrolides tested were azithromycin (AZI, 15 µg) and erythromycin (ERI, 15 µg).

Plates were incubated for 24 h at 37 °C, after which inhibition zone diameters were measured and each isolate was classified as susceptible, intermediate, or resistant according to the appropriate interpretive standards. For *Staphylococcaceae*, breakpoints were applied following CLSI Vet ED07 [23]; when no criteria were available, interpretations followed CLSI M100 ED35 [25], and, if still unavailable, EUCAST guidelines [30]. For *Enterobacteriaceae*, susceptibility classification was based exclusively on CLSI M100 ED34 [24]. Cefoxitin was used as a surrogate for predicting oxacillin resistance results for all *Staphylococcaceae*, except for *S. epidermidis*, in which oxacillin resistance was used as reference, in accordance with CLSI M100 ED34:2024 guidelines. For disk quality control, the strains *Staphylococcus aureus* ATCC 29213 and *Escherichia coli* ATCC 25922 were used. An isolate was classified as MDR if it exhibited non-susceptibility to at least three different antibiotic groups [46]. The categories for each antibiotic used for MDR classification are presented in Supplementary Table 1.

### Molecular Detection of Resistance and Virulence Genes in ***Staphylococcaceae*** and ***E. coli***

#### Polymerase chain reaction (PCR)

The PCR was performed according to [78], with minor modifications. Each 25 µL reaction mixture will contain 2.5 pmol of each primer, 0.2 mM dNTPs, 1.5 mM MgCl₂, 0.6 U of Taq polymerase, and 4.0 µL of bacterial DNA extract. The reaction product, supplemented with 5 µL of loading dye (0.25% bromophenol blue in 50% glycerol), along with a 100 bp molecular marker, was loaded onto a 1.5% agarose gel containing ethidium bromide (1 µg/mL) in Tris–Borate–EDTA buffer and separated by electrophoresis (70 V/1 h).

For *Staphylococcaceae* isolates, detection of the resistance gene *mecA* was performed using the conditions of [76], while genes *sea*, *see*, and *sec* were amplified according to [42]; these targets were chosen based on their clinical relevance and frequent reporting in veterinary and human health context. For *Enterobacteriaceae* isolates, PCR detection of *stx1*, *stx2*, which define the STEC pathotype, and *eae* for EPEC followed the protocol described by [22]. The amplified genes, primers used, positive controls, and sizes of the PCR products are described in Table 1.

**Table 1:** Primer sequences, size of the amplified fragments, positive controls, and references for antimicrobial resistance and virulence genes

Target Genes	Sequence (5’−3’)	Bp	Positive Control	Reference
*Enterobacteriaceae*				
* stx1*	F - AGAGCGATGTTACGGTTTG R - TTGCCCCCAGAGTGGATG	388	Clinical isolate (bovine)	[22]
* stx2*	F - TGGGTTTTTCTTCGGTATC R - GACATTCTGGTTGACTCTCTT	807	ATCC 43895	[22]
* eae*	F - AGGCTTCGTCACAGTTG R – CCATCGTCACCAGAGGA	570	ATCC 43895	[22]
*Staphylococcaceae*				
* mecA*	F-AAAATCGATGGTAAAGGTTGGC R-AGTTCTGCAGTACCGGATTTGC	532	ATCC 43300	[76]
* sea*	F-CCTTTGGAAACGGTTAAAACG R-TCTGAACCTTCCCATCAAAAAC	127	ATCC 13565	[42]
* sec*	F-CTCAAGAACTAGACATAAAAGCTAGG R-TCAAATCGGATTACCATTATCC	271	ATCC 19095	[42]
* see*	F-CTAGTTTGGTAATATCTCCTTTAAACG R-TAACTTACCGTGGACCCTTC	178	ATCC 27664	[42]

#### DNA extraction

DNA extraction from *Enterobacteriaceae* and *Staphylococcaceae* isolates was performed following the method proposed by [9], with minor modifications. Briefly, aliquots of 1.8 mL of bacterial culture from each isolate were centrifuged at 8000 rpm for 5 min at 12 °C to obtain a sufficient cell pellet. The culture medium was discarded, and the bacterial pellet was resuspended in 700 µL of DNA extraction buffer [160 mM Tris-HCl pH 8.0; 60 mM EDTA pH 8.0; 20 mM NaCl; 0.5% (w/v) SDS]. The simultaneous use of lysozyme (for Gram-negative bacteria), mutanolysin (for Gram-positive bacteria with peptidoglycan), and lysostaphin (specifically for staphylococci) was employed to ensure complete and efficient cell wall lysis across the diverse bacterial species analyzed in this study, including both Gram-positive (*Staphylococcaceae*) and Gram-negative (*Enterobacteriaceae*) isolates. This multi-enzyme approach was adapted from the original [9] protocol to accommodate pure bacterial cultures rather than metagenomic samples.

Cell lysis was carried out at 65 °C for 40 min. Then, 300 µL of 5 M potassium acetate solution were added, and after homogenization, the mixture was kept on ice for 30 min. Purification was performed using 600 µL of chloroform: isoamyl alcohol (24:1, v/v) followed by centrifugation at 12,000 rpm for 10 min at 10 °C. The clear supernatant was transferred to new tubes, to which 1,000 µL of ice-cold absolute ethanol were added. The solution was gently mixed and stored at − 20 °C for 12 h to allow DNA precipitation. Afterwards, tubes were centrifuged at 12,000 rpm for 17 min at 10 °C to obtain the DNA pellet. The supernatant was discarded, and the pellet was washed with 700 µL of 70% (v/v) ethanol, dried at 55 °C, and resuspended in 30–50 µL of TE buffer (10 mM Tris-HCl pH 8.0; 1 mM EDTA pH 8.0).

DNA concentrations and purity were determined using a NanoDrop One spectrophotometer (Thermo Scientific, Waltham, USA).

## Results

Out of 120 culture plates (60 on EMB and 60 on MSA, derived from the same 60 fly samples), 34 distinct colonies grown and were successfully identified (17 *Enterobacteriaceae* and 17 *Staphylococcaceae*). Colonies growth on both agars was observed in 6 samples (10%), all originating from meat processing industries. No *Enterobacteriaceae* were recovered from the swine slaughterhouse. The proportion of positive samples (*Enterobacteriaceae* or *Staphylococcaceae*) was highest in flies from meat processing industries (15/29, 51.7%), followed by bovine slaughterhouses (11/26, 42.3%) and swine slaughterhouses (2/5, 40%).

**Fig. 1:**
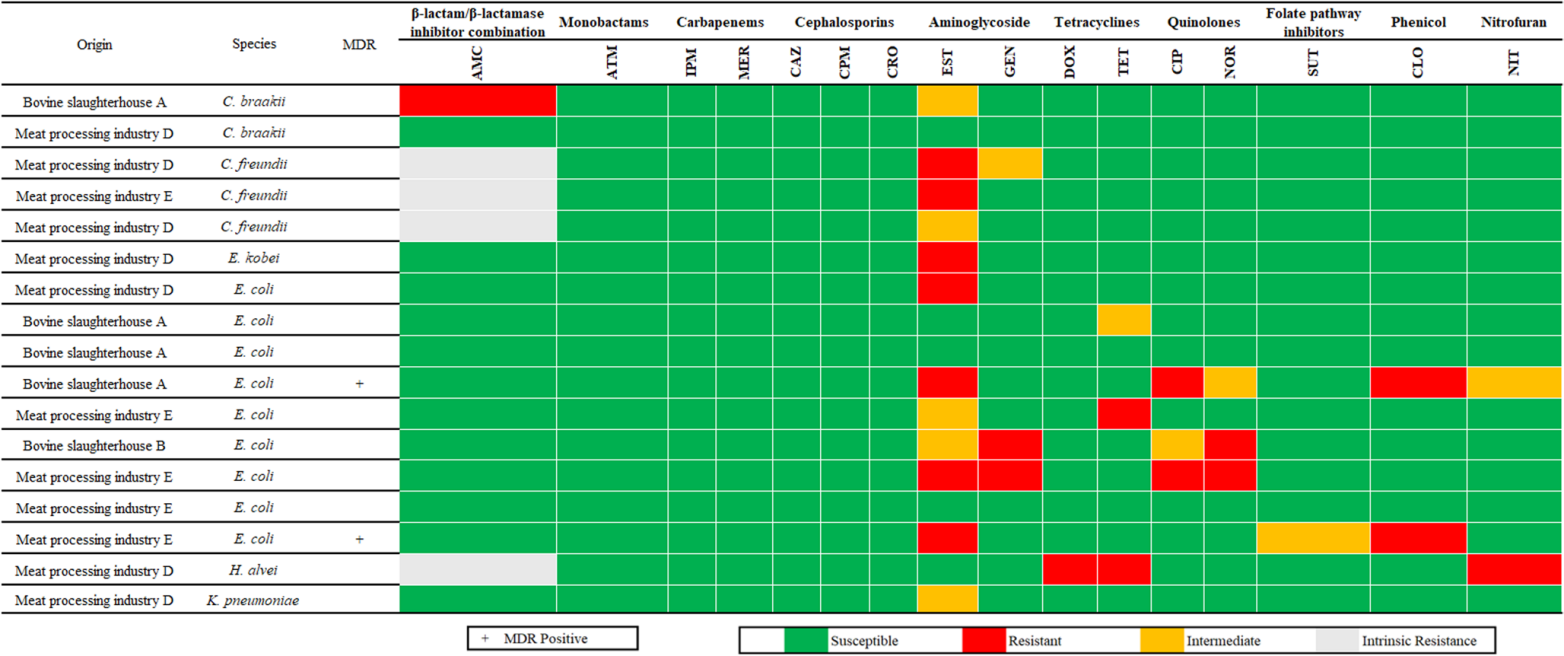
Identification results and AMR profiles of bacterial isolates with a metallic green sheen on EMB agar. Species were identified using MALDI-TOF mass spectrometry, and their classification as MDR is included. The figure also details the sample origin and the resistance profiles for 16 antibiotics from 10 distinct antibiotic groups. Both *Hafnia alvei* and *Citrobacter freundii* are intrinsically resistant to amoxicillin-clavulanic acid according to CLSI (2024). AMC—Amoxicillin + Clavulanic Acid, ATM—Aztreonam, CAZ—Ceftazidime, CIP—Ciprofloxacin, CLO—Chloramphenicol, CPM—Cefepime, CRO—Ceftriaxone, DOX—Doxycycline, EST—Streptomycin, GEN—Gentamicin, IPM—Imipenem, MER—Meropenem, NIT—Nitrofurantoin, NOR—Norfloxacin, SUT—Sulfamethoxazole + Trimethoprim, TET—Tetracycline

A set of 17 isolates that exhibited a metallic green sheen on EMB agar were obtained and subsequently identified using MALDI-TOF MS. Not all of the isolates were identified as *E. coli*: there were nine isolates of *E. coli*, three isolates of *Citrobacter freundii*, two isolates of *Citrobacter braaki*, one isolate of *Enterobacter kobei*, one isolate of *Klebsiella pneumoniae* and lastly one isolate of *Hafnia alvei*. Two *E. coli* isolates were classified as MDR (11.8%), one exhibited resistance to streptomycin, ciprofloxacin and chloramphenicol while the other was resistant to streptomycin, chloramphenicol and non-susceptible to sulfamethoxazole with trimethoprim (Fig. 1). The highest number of resistant isolates was observed for streptomycin (7 isolates, including *C. freundii*, *E. kobei* and *E. coli*).

**Fig. 2:**
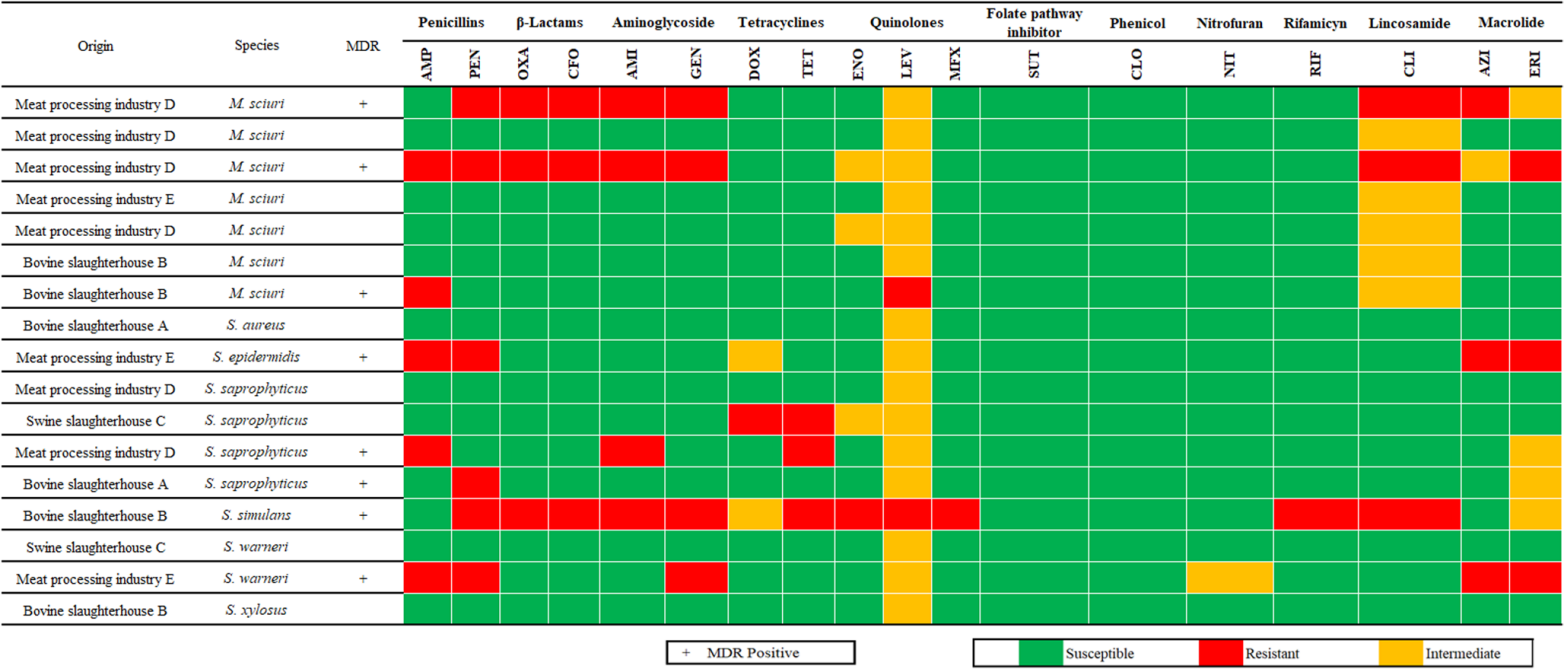
Identification results and AMR profiles of bacterial isolates in MSA. Species were identified using MALDI-TOF mass spectrometry, and their classification as MDR is included. The figure also details the sample origin and the resistance profiles for 18 antibiotics from 11 distinct antibiotic groups. Cefoxitin disk diffusion result was used as a surrogate for predicting oxacillin resistance for all *Staphylococcaceae*, except for *S. epidermidis*, in which oxacillin resistance was used as the reference, in accordance with CLSI (2024) guidelines. AMI—Amikacin, AMP—Ampicillin, AZI—Azithromycin, CFO—Cefoxitin, CLI—Clindamycin, CLO—Chloramphenicol, DOX—Doxycycline, ENO—Enrofloxacin, ERI—Erythromycin, GEN—Gentamicin, LEV—Levofloxacin, MFX—Moxifloxacin, NIT—Nitrofurantoin, OXA—Oxacillin, PEN—Penicillin, RIF—Rifampicin, SUT—Sulfamethoxazole + Trimethoprim, TET—Tetracycline

A set of 17 *Staphylococcaceae* were obtained and subsequently identified. There was one *Staphylococcus aureus*, one *Staphylococcus epidermidis*, four *Staphylococcus saprophyticus*, seven *Mammaliicoccus sciuri*, one *Staphylococcus simulans*, two *Staphylococcus warneri* and one *Staphylococcus xylosus*. We found eight MDR isolates (47.1%). Notably, the single *S. simulans* isolate was resistant to seven groups of antibiotics. Figure 2 shows the resistance profiles of the *Staphylococcaceae* isolates and their origins.

AMR isolates were detected in flies from all five sampled facilities. MDR strains were identified in both bovine slaughterhouses (A and B), with two MDR isolates each, and in both meat processing industries (D and E), with three MDR isolates each.

The virulence genes *stx1*, *stx2* and *eae* were not detected in any of the *E. coli* isolates. Similarly, the *mecA*, *sea*, *sec* and *see* genes were absent from all *Staphylococcus* and *Mammaliicoccus sciuri* isolates.

## Discussion

Bacteria isolated from flies in food production environments are of particular concern because they may act as reservoirs and disseminator of antimicrobial resistant strains, thereby posing risks to food safety and public health. The diversity of *Enterobacteriaceae* and *Staphylococcaceae* species recovered from the fly samples reflect the broad spectrum of bacterial taxa that can be carried by these insects in meat production environments. It is important to note that different genera of *Enterobacteriaceae* are capable of producing the metallic green sheen typically associated with *E. coli* on EMB agar [28]. This information explains the variety of species recovered in our study from EMB agar plates and highlights the ecological overlap among enteric bacteria disseminated by flies.

A total of nine isolates were identified as *E. coli* and they exhibited variable AMR profiles. Two isolates (22.2%) were MDR, two isolates (22.2%) were susceptible to all tested antibiotics, while four (44.4%) showed resistance to streptomycin. These values are lower than those reported by [3], who observed 88% of *E. coli* resistant to streptomycin, and [71], who reported 87.4% resistance in fly isolates. *E. coli* is a major opportunistic pathogen capable of causing a wide range of human infections, including urinary tract infections, respiratory tract infections and bloodstream infections [40]. The presence of MDR *E. coli* strains, as observed in two of the study’s isolates, further complicates these infections, as resistance can limit therapeutic options and increase both morbidity and mortality [29].

Other *Enterobacteriaceae* were less frequent but still noteworthy. *C. braakii* and *C. freundii* were isolated in lower frequency. Although resistance was limited in our samples, previous studies have reported highly resistant clinical and fly-derived isolates, including carbapenemase-producing strains [27, 85, 88]. Although isolated only once, and with minimal resistance, *K. pneumoniae* and *H. alvei* are notable due to clinical significance and MDR strains in other contexts [38, 66]. To our knowledge, this study provides the first report of *E. kobei* isolated from flies. Its detection in meat production environments broadens the recognized ecological niche of this species and underscores the role of flies as potential disseminators of clinically relevant opportunistic pathogens. Collectively, the *Enterobacteriaceae* isolated in this study highlights the role of flies as carriers of opportunistic bacteria which may serve as reservoirs capable of acquiring and transmitting important antimicrobial resistance traits under appropriate selective pressures.

The family *Staphylococcaceae* was also diverse in our samples, with several species recovered in low frequency but carrying relevant antimicrobial resistance profiles. Only a single isolate of *S. aureus* (1.7%) was identified, and it was not resistant to any antimicrobials. Although this frequency in flies is considerably lower than the 78.6% reported by [3], it is more consistent with the 13.8% observed by [59]. *S. aureus* remains of major concern given its clinical relevance, broad pathogenic potential, and the emergence of methicillin-resistant strains mediated by the *mecA* gene [4, 70]. Other coagulase-negative staphylococci were more frequent, and some showed relevant resistance traits. *S. epidermidis* (1.7%) displayed macrolide and penicillin resistance, consistent with its role as an AMR gene reservoir, although *mecA* was not detected [31]. *S*. *saprophyticus* (6.7%), which can cause urinary tract infections [44], was recovered in four isolates, with two classified as MDR.

The most prevalent *Staphylococcaceae* species was *Mammaliicoccus sciuri* (11.7%), with two MDR isolates resistant to penicillins, β-lactams, aminoglycosides, lincosamides, and macrolides. Although often opportunistic, *M. sciuri* has been linked to severe infections, including fatal pneumonia with sepsis and acute respiratory distress syndrome in companion animals [68]. Virulence and resistance genes from *M. sciuri* can transfer to other *Staphylococcus* species in the same habitat [52]. Although our isolates lacked *mecA*, their resistance profiles emphasize the relevance of *M. sciuri* in One Health dynamics and the potential role of flies in disseminating AMR in meat production environments.

Less frequent but relevant species included *S. simulans* (1.7%), which showed resistance to most tested antimicrobial classes, remaining susceptible only to folate pathway inhibitors, phenicols, nitrofurans. Besides its role in bovine subclinical mastitis [82], it has been also associated in with human illness as well [75]. *S. warneri* (3.3%) comprised of one MDR isolate and another non-susceptible only to levofloxacin, while the single *S. xylosus* (1.7%) isolate showed the same profile, consistent with its commensal presence on animal skin and mucosal surfaces.

When resistance profiles were compared between bacterial groups, *Enterobacteriaceae* generally showed lower resistance levels than *Staphylococcaceae*, a pattern also reflected in the differences in MDR prevalence reported by [56]. Notably, none of the *Enterobacteriaceae* isolates in our study were resistant to cephalosporins, in contrast to the very high resistance rates (exceeding 80%) to third-generation cephalosporins in isolates recovered from flies in China [85]. This discrepancy suggests that flies in the industries sampled by this study may not yet represent a significant reservoir of *Enterobacterales* resistant to third-generation cephalosporins and potential producers of extended-spectrum beta-lactamases (ESBL) [57].

In flies collected from the swine slaughterhouse, *Staphylococcaceae* were recovered from 40% of samples, a prevalence similar to the 37.3% reported by [13, 14] in swine farms. In contrast, no *Enterobacteriaceae* were isolated from swine slaughterhouse flies in our study, whereas [45] reported *E. coli* in 91.5% of fly samples from pig farms. Similarly, [56] detected *E. coli* in 50% and *S. aureus* in 25% of slaughterhouse fly samples, compared with 15% and 1.7%, respectively, in our study. These differences highlight the substantial variation in bacterial carriage in flies across distinct steps of meat production chains and geographic contexts, underscoring the need for localized surveillance to better understand the epidemiological role of flies in antimicrobial resistance dissemination.

Oxacillin resistance was detected in three (17.6%) staphylococcal isolates, a concerning finding given its classification as a critically important antimicrobial [84]. Notably, phenotypic resistance occurred in the absence of *mecA*, the main determinant of oxacillin resistance, suggesting alternative mechanisms. Similar genotype–phenotype mismatches have been reported elsewhere [35, 62], potentially explained by homologous genes such as *mecC*, which may escape detection by *mecA*-targeted primers [10]. Clinically, this is relevant since methicillin/oxacillin resistance in staphylococci is often linked to resistance to multiple other antibiotic classes, reducing therapeutic options and increasing the risk of treatment failure [1]. The prevalence oxacillin resistance in our study was higher than the rates reported for *S. aureus* by [59] in South Africa (1.5%). This discrepancy may be attributed to the diversity of species in our cohort, which included other resistant *Staphylococcaceae* such as *M. sciuri* and S. *simulans*, suggesting that the overall burden of oxacillin resistance in flies may be underestimated when focusing solely on *S. aureus*. The clinical importance of β-lactams resistance, such as oxacillin, cannot be overstated, as β-lactam antibiotics represent the cornerstone of antimicrobial therapy for staphylococcal infections [43]. Multiple recent studies have shown the role of flies in disseminating β-lactamase-producing bacteria, being able to harbor and transmit β-lactam-resistant strains between farm environments, processing facilities, and ultimately to human populations [15, 47, 83].

The issue of MDR also presents an interesting comparison. Our study found ten MDR isolates, a prevalence of 16.7% from the total samples and 29.4% from the total isolates. This is higher than the 5.9% of flies carrying MDR strains reported by [19] in Brazil, and lower than the 32.5% MDR prevalence in flies noted by [13, 14] in Italy. Our finding is significantly lower than the 100.0% MDR rate for *E. coli* fly isolates reported by [71] and [85]. This variation in MDR prevalence may be influenced by the different environments from which samples were collected, the bacterial species studied, and antimicrobial usage in the surrounding areas. Despite the low number of MDR isolates in our study, their presence remains concerning, as contaminated flies can disseminate these bacteria across multiple niches within the production chain and to workers and animals in the surrounding environment, thereby increasing the risk of occupational exposure and animal colonization [54, 74]. Our research on virulence genes found that all isolates tested for *stx1*, *stx2*, and *eae* were negative. This is in agreement with [73], who also found no presence of *stx1* and *stx2* in their *E. coli* isolates. Furthermore, the absence of the *mecA* gene in our *Staphylococcaceae* isolates is consistent with [48], who did not detect this gene in their fly samples. However, this contrasts with [3], who found that 14% of their penicillin and amoxicillin-resistant *S. aureus* isolates were *mecA* positive. The lack of these virulence genes in our isolates is a positive finding but the potential role of flies in transmitting AMR and virulent strains should not be underestimated.

In our study, flies from meat processing industries carried more bacterial isolates than those from bovine and swine slaughterhouses. This may reflect facility characteristics, as processing plants involve more steps in the production chain, increasing opportunities for cross-contamination of products, surfaces, and staff [67]. Location may also play a role: slaughterhouses were in rural areas, while processing facilities were urban, where flies encounter denser human activity and additional microbial sources such as sewage. These factors together highlight the need for targeted vector control especially in meat processing industries to limit bacterial dissemination along the food chain.

In Brazil, flies have been repeatedly shown to act as vectors of antimicrobial-resistant bacteria in diverse environments. In peri-hospital settings in Rio de Janeiro, [17] detected resistance genes in *Klebsiella quasipneumoniae subsp. similipneumoniae*, while [18] reported an *E. coli* isolate carrying *bla*_NDM−1_. A broader survey of 117 flies near hospitals found that 35.9% carried resistant bacteria and 5.9% harbored MDR strains [19]. In food-production systems, [6] recovered 198 *E. coli* isolates from flies in São Paulo dairy farms, with over 30% classified as MDR, while [7] reported polymyxin resistance in 33.3% of *E. coli* from flies. Similarly, [5] identified *E. coli*, *Salmonella* spp., and *Staphylococcus* spp. from synanthropic flies in dairy farms in Paraná, Brazil. Collectively, these studies confirm the role of flies in disseminating AMR in Brazil. To our knowledge, however, this is the first study to investigate AMR in bacterial isolates from flies in the meat production chain, expanding the understanding of their ecological role as vectors of resistant bacteria.

Future studies should broaden investigations of flies as reservoirs and disseminators of bacteria in meat production. Wider molecular screening of resistance and virulence genes would better characterize their genetic potential and may explain the observed phenotype-genotype discordance. Longitudinal designs across seasons and production stages could clarify temporal dynamics, while comparative approaches in different regions and processing steps would show how local practices influence dissemination. Such research would refine understanding of the epidemiological role of flies and guide targeted hygiene and vector control measures in food production. Future investigations should also incorporate morphological or molecular identification of fly species to better characterize the specific vectors involved in AMR dissemination within meat processing chains and to enable targeted vector control strategies based on species-specific biology and behavior.

This study has limitations that should be taken into account. An important methodological consideration relates to the interpretation of oxacillin resistance among all *Staphylococcaceae*, except for *S. epidermidis*. Cefoxitin disk diffusion was used exclusively as a surrogate marker for predicting oxacillin resistance, in accordance with CLSI M100 ED34:2024 recommendations, and should not be interpreted as evidence of generalized resistance to β-lactam antibiotics as a class. The performance of cefoxitin disk diffusion is known to be heterogeneous among coagulase-negative staphylococci and closely related genera, with variable sensitivity and specificity depending on the species, and reduced predictive accuracy compared with *S. aureus* [64]. In the absence of minimum inhibitory concentration determination, cefoxitin-based interpretation allows inference of resistance to oxacillin only and does not validate resistance to other β-lactam subclasses. Therefore, while the phenotypic findings reported here provide valuable baseline information, confirmatory testing using MIC-based methods or molecular assays will be essential in future studies to refine resistance characterization and strengthen epidemiological interpretation. The restricted sample size and geographic scope may limit generalizability to other regions or production systems. Another important limitation of this study is the absence of taxonomic identification of fly species. Different dipteran species exhibit distinct ecological behaviors, habitat preferences, and vectorial capacities that directly influence their role in bacterial dissemination. In addition, an important limitation of this study is the restricted genetic panel, which included only seven genes (*mecA*, *sea*, *see*, *sec*, *stx1*, *stx2*, *eae*). This limited screening likely explains the absence of molecular confirmation for phenotypically resistant isolates. Despite these constraints, the study provides valuable insight into the potential of flies to disseminate MDR bacteria in meat production environments and highlights areas for further research.

## Conclusion

This study demonstrates that flies present in Brazilian slaughterhouses and meat-processing industries can carry bacteria of public-health relevance, including 13 species across *Enterobacteriaceae* and *Staphylococcaceae*, with ten isolates classified as MDR (two *E. coli*, one *S. epidermidis*, two *S. saprophyticus*, one *S. simulans*, one *S. warneri* and three *M. sciuri*). Notably, oxacillin resistance was detected in 17.6% of *Staphylococcaceae* isolates and streptomycin resistance in 41.2% of *Enterobacteriaceae*, despite the absence of the targeted resistance and virulence genes (*mecA*, *sea*, *see*, *sec*, *stx1*, *stx2*, *eae*). These findings indicate that flies contribute to the dissemination of phenotypically resistant bacteria within meat production environments, reinforcing their relevance from a One Health perspective.

The interpretation of these results is constrained by the sample size, the limited gene panel screened, and the absence of fly-species identification, which could influence bacterial carriage profiles. Even so, the consistent recovery of resistant isolates across different facilities highlights the need for enhanced hygiene protocols, routine AMR surveillance, and targeted fly-control strategies to reduce microbial spread along the meat production chain.

## Supplementary Information

Below is the link to the electronic supplementary material.

Supplementary Material 1

## Data Availability

The datasets generated during and/or analyzed during the current study are available from the corresponding author on reasonable request.
