# Supplementary material for: Flies from meat processing facilities are carriers of multidrug-resistant Escherichia coli and diverse Staphylococcaceae species
Source: Braz J Microbiol. 2026 Feb 23;57(1):60. doi: 10.1007/s42770-026-01883-2 (PMC12926286; doi:10.1007/s42770-026-01883-2)
Supplement: Supplementary file 1 — Supplementary Material 1 [file 42770_2026_1883_MOESM1_ESM.docx]

**Table S1** Antibiotics tested and their corresponding categories. Isolates non-susceptible to three or more categories were classified as multidrug resistant.

| Category | Antibiotic |
| --- | --- |
| β-lactam/β-lactamase inhibitor combination | Amoxicillin + Clavulanic Acid |
| β-Lactams | Oxacillin |
|  | Cefoxitin |
| Aminoglycoside | Amikacin |
|  | Gentamicin |
|  | Streptomycin |
| Carbapenems | Imipenem |
|  | Meropenem |
| Cephalosporins | Ceftazidime |
|  | Cefepime |
|  | Ceftriaxone |
| Folate pathway inhibitor | Sulfamethoxazole + Trimethoprim |
| Lincosamide | Clindamycin |
| Macrolide | Azithromycin |
|  | Erythromycin |
| Monobactams | Aztreonam |
| Nitrofuran | Nitrofurantoin |
| Penicillins | Ampicillin |
|  | Penicillin |
| Phenicol | Chloramphenicol |
| Quinolones | Enrofloxacin |
|  | Levofloxacin |
|  | Moxifloxacin |
|  | Ciprofloxacin |
|  | Norfloxacin |
| Rifamicyn | Rifampicin |
| Tetracyclines | Doxycycline |
|  | Tetracycline |
